# Massive Incidental Pneumoperitoneum in an Amyotrophic Lateral Sclerosis Patient

**DOI:** 10.7759/cureus.18678

**Published:** 2021-10-11

**Authors:** Jasmin Jaber, Nur Magadle, Lojain Arda, Francisco J Somoza-Cano

**Affiliations:** 1 Internal Medicine, Hadassah Ein Kerem, Jerusalem, ISR; 2 Internal Medicine, St. Vincent Charity Medical Center, Cleveland, USA

**Keywords:** iatrogenic complication, percutaneous endoscopic gastrostomy tube, desaturation, pneumoperitoneum, amyotrophic lateral sclerosis

## Abstract

In this report, we present the case of a 61-year-old male patient diagnosed with amyotrophic lateral sclerosis (ALS), who presented to the ER with worsening shortness of breath, several hours following elective percutaneous endoscopic gastrostomy (PEG) tube placement. During his hospitalization, he was diagnosed with massive pneumoperitoneum, a potential complication of such procedures. We aim to provide a general overview of this condition and to discuss the special considerations in the treatment of ALS.

## Introduction

Amyotrophic lateral sclerosis (ALS), or Lou Gehrig’s disease, is a progressive neurodegenerative disorder affecting upper motor neurons in the motor cortex and lower motor neurons in the brainstem and spinal cord. It was first described by Jean-Martin Charcot in the 19th century, who attempted to differentiate it from other neurological pathologies [1].

ALS affects one to two individuals per 100,000 population per year in the US and Europe. Approximately 10% of all ALS cases are familial; the remaining 90% are sporadic, and pathology between the two forms is identical. Disease onset usually occurs in the fifth decade of life, with earlier onset indicating a familial pattern. The disease is characterized by progressive muscle weakness and disability [2], as well as malnutrition due to dysphagia and hypermetabolism [3].

Due to its insidious onset, the time to diagnosis is approximately 12 months, with death usually occurring due to respiratory failure within three to five years of diagnosis. Percutaneous endoscopic gastrostomy (PEG) tube placement in ALS patients is associated with improved nutrition and decreased mortality rates, if performed early in the disease course [4].

## Case presentation

A 61-year-old male presented with hypoxia and worsening shortness of breath, several hours after a PEG tube placement. His medical history included ALS diagnosed four years prior, for which he had received riluzole.

On admission, the patient was tachypneic (up to 40 breaths per minute) and tachycardic (135 beats per minute); oxygen saturation on room air was 85% but improved to 95% with a face mask. On physical examination, decreased air entry was noted in the left lower lung field with diffuse rhonchi. The PEG was noted to be in place with mild erythema and localized tenderness, and no discharge was noted from the wound site. The abdomen was soft and lax.

**Figure 1:**
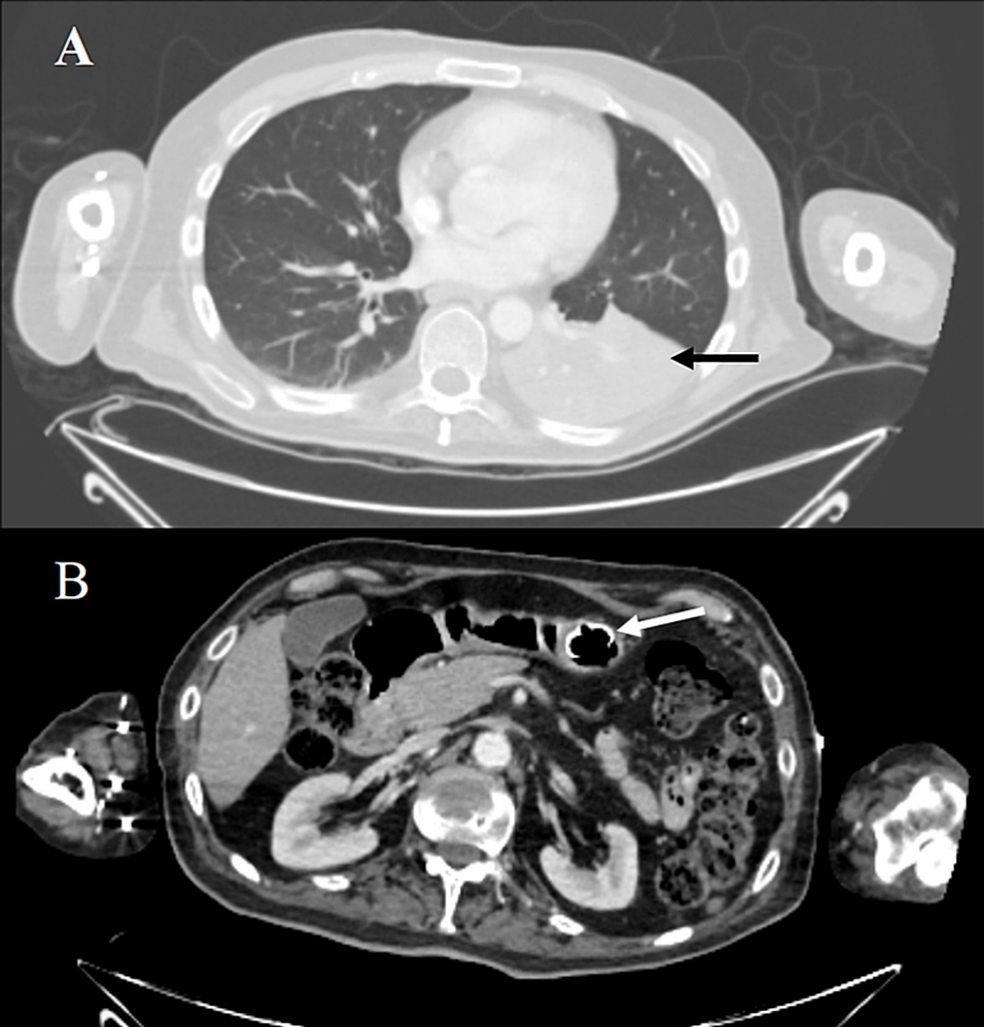
Chest and abdominal CT A. Chest CT showing LLL consolidation with obstruction of the accompanying bronchus with fluid content (black arrow). B. Abdominal CT with PEG tube in place with no evidence of leak or free air (white arrow) CT: computed tomography; LLL: left lower lobe; PEG: percutaneous endoscopic gastrostomy

Initial blood tests revealed a mild leukocytosis (20.6/microliter); C-reactive protein (CRP) was 0.2 mg/dL. Electrolytes, renal, and liver functions were within normal levels. He underwent a CT scan of his chest, abdomen, and pelvis, revealing a left lower lobe consolidation with obstruction of the accompanying bronchus by fluid contents. The PEG tube was noted to be in place (Figure 1). Due to the suspicion of aspiration pneumonitis, empiric antibiotics and intravenous fluids were started, and he was admitted to the internal ward.

**Figure 2:**
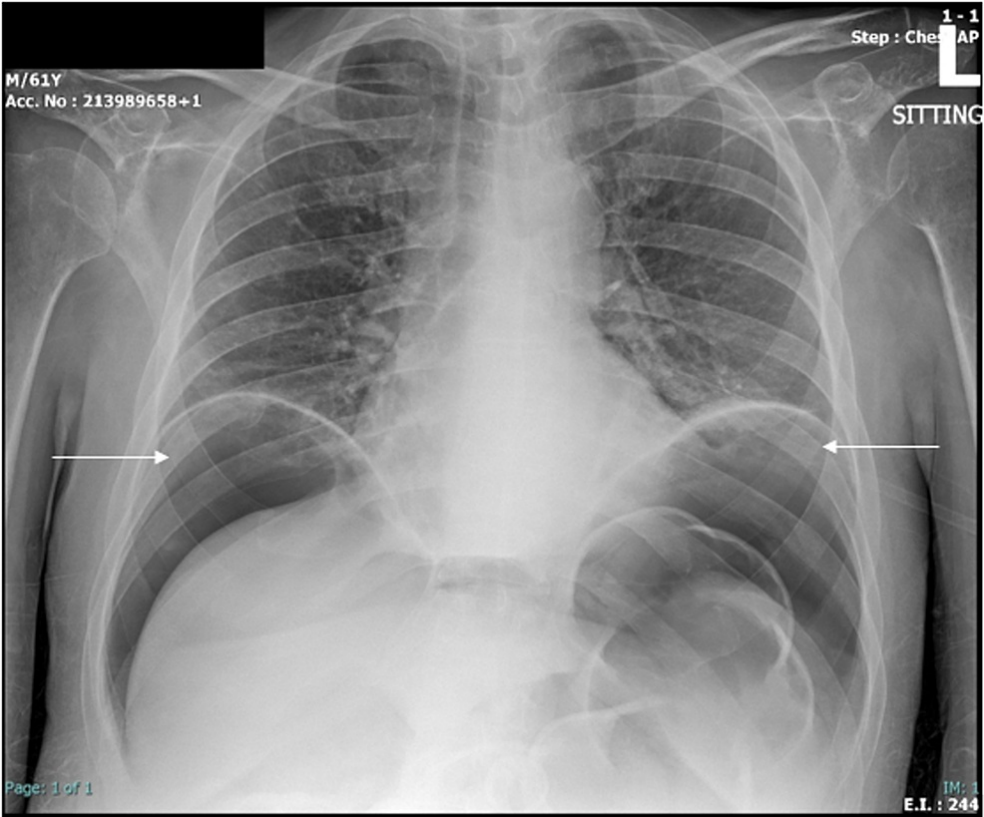
AP chest X-ray showing large amounts of subdiaphragmatic free air, bilaterally (arrows) AP: anteroposterior

The following day, approximately 28 hours after the admission, a chest radiograph was performed for follow-up, revealing large amounts of free air under the diaphragm bilaterally (Figure 2). On repeat examination, vital signs were within normal limits, and the patient was in no acute distress, with normal O_2_ saturation on nasal cannula. The abdomen was soft and non-tender.

**Figure 3:**
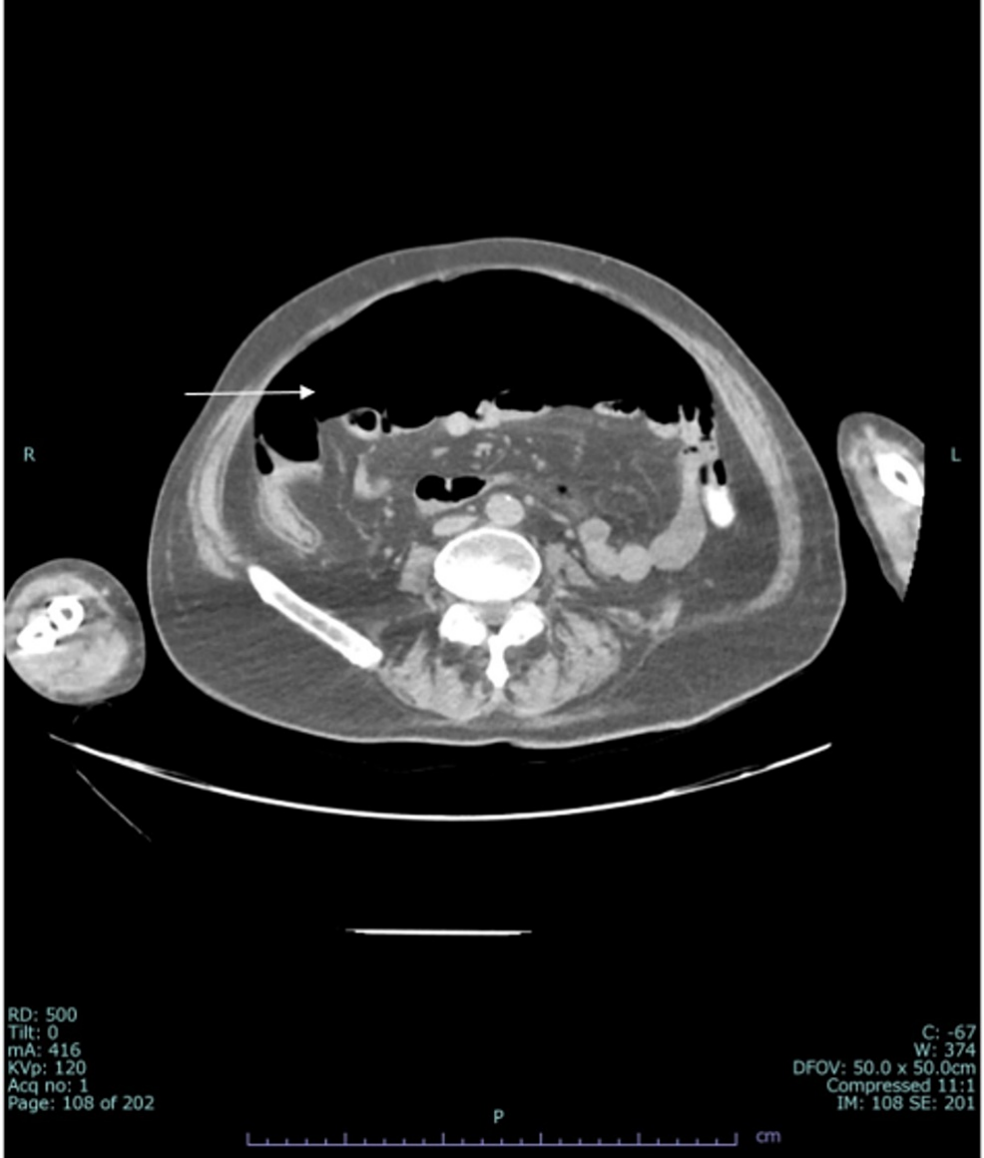
Axial CT of the abdomen showing large pneumoperitoneum (arrow) CT: computed tomography

A repeat CT scan revealed massive intraperitoneal free air but no signs of hollow organ perforation (Figure 3). PEG tube was again found to be in place. Following surgical consultation, the tube remained unused for 48 hours, with a periodic opening to allow air drainage. Under close observation, the patient remained clinically stable; thereafter, fluids and medication were given by PEG without any immediate complications, and tube feeding was started. The patient was discharged in a stable condition.

## Discussion

ALS is a progressive neurodegenerative disease affecting the upper and lower motor neurons. The disease is strongly associated with high mortality, typically due to respiratory failure or complications such as dysphagia.

PEG placement is a safe and widely used means of providing enteral feeding, with pneumoperitoneum representing a benign and self-limiting outcome. However, in some cases, it may be a sign of a perforated hollow viscus [5]. Several studies have been conducted to assess the incidence and clinical significance of pneumoperitoneum after PEG insertion (in both ALS and non-ALS patients), with relatively small sample sizes [6,7]. In other studies, CT was found to be more sensitive in diagnosing free air than upright chest radiography, especially in patients without clinical signs or symptoms [8,9]. A retrospective chart review conducted by Blum et al. [10] involving 722 patients reported a 12% incidence of free intra-abdominal air following PEG placement.

Unusual clinical presentation and low clinical suspicion often lead to a delay in diagnosis and, consequently, inappropriate therapy. While serious complications following PEG placement are rare, injuries can be disastrous and may require urgent surgical intervention.

## Conclusions

We presented the case of a 61-year-old patient with massive, asymptomatic, and iatrogenic pneumoperitoneum. While being a recognized complication of PEG insertion in all patients, the risk of procedural complications in patients with musculoskeletal or nervous system disorders is more significant. Clinicians should take into account underlying medical conditions and be aware of potential red flags, including fever, altered mental status, or signs of peritonitis. Patients presenting with seemingly benign moderate or large pneumoperitoneum after an invasive procedure should not be disregarded and should be closely monitored, especially in the first 24 hours.
